# The impact of eliminating primary school tuition fees on child marriage in sub-Saharan Africa: A quasi-experimental evaluation of policy changes in 8 countries

**DOI:** 10.1371/journal.pone.0197928

**Published:** 2018-05-24

**Authors:** Alissa Koski, Erin C. Strumpf, Jay S. Kaufman, John Frank, Jody Heymann, Arijit Nandi

**Affiliations:** 1 UCLA Fielding School of Public Health, Los Angeles, California, United States of America; 2 Department of Economics, McGill University, Montreal, Quebec, Canada; 3 Department of Epidemiology, Biostatistics, and Occupational Health, McGill University, Montreal, Quebec, Canada; 4 Scottish Collaboration for Public Health Research and Policy, Usher Institute of Population Health Sciences and Informatics, University of Edinburgh, Edinburgh, United Kingdom; 5 Institute for Health and Social Policy, McGill University, Montreal, Quebec, Canada; Arizona State University, UNITED STATES

## Abstract

**Background:**

Child marriage harms girls’ health and hinders progress toward development goals. Randomized studies have shown that providing financial incentives for girls’ education can effectively delay marriage, but larger-scale interventions are needed in light of slow progress toward curbing the practice. Many sub-Saharan African countries eliminated primary school tuition fees over the past two decades, resulting in massive increases in enrolment. We measured the effect of these policies on the probability of primary school completion and of marriage before 15 and 18 years of age.

**Methods:**

We used Demographic and Health Surveys to assemble a dataset of women born between 1970 and 2000 in 16 countries. These data were merged with longitudinal information on the timing of tuition fee elimination in each country. We estimated the impact of fee removal using fixed effects regression to compare changes in the prevalence of child marriage over time between women who were exposed to tuition-free primary schooling and those who were not.

**Results:**

The removal of tuition fees led to modest average declines in the prevalence of child marriage across all of the treated countries. However, there was substantial heterogeneity between countries. The prevalence of child marriage declined by 10–15 percentage points in Ethiopia and Rwanda following tuition elimination but we found no evidence that the removal of tuition fees had an impact on child marriage rates in Cameroon or Malawi. Reductions in child marriage were not consistently accompanied by increases in the probability of primary school completion.

**Conclusions:**

Eliminating tuition fees led to reductions in child marriage on a national scale in most countries despite challenges with implementation. Improving the quality of the education available may strengthen these effects and bolster progress toward numerous other public health goals.

## Introduction

Child marriage is a threat to girls’ health and to development across the globe. Married girls are more likely to experience violence at the hands of their husbands and have less control over their reproductive lives [1]. They begin childbearing earlier, have less access to contraception, give birth at shorter intervals, and report having more unintended pregnancies when compared to their peers who marry after the age of 18 [2,3] They are also at greater risk of acquiring sexually transmitted infections, including HIV [4,5]. All of these outcomes are associated with increased risk of obstetric complications, the leading cause of death among young women in low-and middle-income countries [6]. In addition to the health risks associated with the practice, married girls have fewer years of schooling than their peers who wed later, potentially diminishing long-term economic opportunities for themselves and their families [7–9]. The harmful consequences of child marriage may be more severe among girls younger than 15 relative to older adolescents as a result of their greater physiological immaturity.

Sub-Saharan Africa has the highest rates of child marriage in the world. More than one in three girls marry before their 18^th^ birthday throughout much of the region. Although marriage among older adolescents has declined over the past twenty years, recent estimates indicate that little progress has been made toward reducing marriage among girls under 15 years of age [10].

Eliminating child marriage is one of the targets for achieving United Nations Sustainable Development Goal 5: gender equality and the empowerment of all women and girls. Meeting this target will require focusing resources on interventions proven to delay marriage. Two recent systematic reviews of programs aimed at preventing child marriage in low-income countries found that providing various financial incentives for girls’ schooling are the only interventions that have been quantitatively shown to delay marriage [11,12]. Although a variety of other interventions have been fielded, very few have been rigorously evaluated [11].

Some of the strongest evidence supporting reduced education costs as a mechanism for delaying marriage comes from randomized studies conducted in sub-Saharan Africa. In Zomba District, Malawi, providing monthly cash transfers to girls between 13 and 22 years of age reduced the odds of marriage after one year of follow-up by 52% among those who were not enrolled in school at baseline and by 32% among girls who were enrolled at baseline, though the latter estimate was imprecise and not statistically significant [13]. Hallfors et al. (2015) enrolled orphaned girls in Manicaland, Zimbabwe in a randomized trial that provided large school subsidies including tuition fees, uniforms, supplies, and a “helper” to monitor school attendance. The girls were in Grade 6 at baseline and 12 years of age, on average. Five years later, when the girls were approximately 17 years old, the odds of marriage were 63% lower among girls who received the subsidy relative to those in the control arm of the trial [14]. Girls in two districts in Western Kenya who received free school uniforms when they were in Grade 6 were 2.6 percentage points less likely to be married three years later and 3.9 percentage points less likely seven years later, when they were 20.5 years of age, on average [15].

Although these studies provide high-quality evidence, the extent to which the findings can be generalized is unclear due to the limited geographical areas in which they were implemented and the potential of the interventions for scale up is unknown. In light of the slow progress toward eliminating child marriage in sub-Saharan Africa in recent decades, larger scale interventions will be necessary to curb the practice and to meet the Sustainable Development Goal target by 2030.

Since the mid-1990s many countries in the region have eliminated tuition fees for public primary schooling in response to renewed international efforts to achieve universal primary education. The policies had a shared objective across the countries–to improve access to primary schooling–and received a high level of political support. Tuition fee elimination was part of broader education reform in many countries, the scope and substance of which varied, but dramatic increases in enrolment followed in every case [16].

These policies represent a significant reduction in the direct costs of schooling. Attending school may involve other direct and indirect costs to families, such as additional fees for uniforms, exams, and the loss of the child’s labor, but the elimination of tuition fees may lower barriers to enrollment for many students from poor families, particularly girls [17]. The policies could have delayed marriage through two main pathways. First, girls who were previously unable to enroll or whose schooling was curtailed due to untenable expense may increase the duration of their schooling in response to the elimination of tuition fees. This could delay marriage if girls are considered unavailable while they are actively attending school. Second, greater learning and social interaction may empower girls to resist early marriage.

Very few studies have rigorously examined the downstream effects of these policies [18]. A recent systematic review of the effects of eliminating school fees in low-income countries identified a single quasi-experimental study that estimated the effect of tuition-fee removal in Uganda on the age at which children enrolled in primary school [19,20]. Osili and Long (2008) exploited geographical variation in program intensity to measure the effect of tuition fee removal in Nigeria in 1976 and found that the policy change led to a significant increase in the duration of schooling and a decrease in fertility [21].

One reason for the limited number of impact evaluations may be the fact that in most cases the policies were implemented at a single point in time across the entire country, thereby limiting sources of variation in exposure and restricting options for valid comparison within countries. We make use of the similarity in policies implemented across many countries within the region to estimate their effect on the prevalence of marriage before 15 and 18 years of age across a pooled sample from 16 countries as well as the effect within individual countries. Since increased educational attainment is the most likely pathway through which the policies could affect child marriage, we also estimated the impact of the policies on the probability of completing primary school.

## Methods

### Data sources

We identified sixteen countries in sub-Saharan Africa that adopted policies intended to eliminate primary school tuition fees and for which we were able to reliably establish when the policies took effect (Table 1). This information was obtained from reports on the education systems in each country submitted to the UNESCO International Bureau of Education and corroborated by reports from the World Bank [16,18]. The same sources provided information on the structure of public schooling systems in each country, including the expected age at enrollment and duration of primary school.

**Table 1:** The year primary school tuition fees were eliminated, the expected age at primary school enrollment, and the earliest birth cohort exposed in each country.

Country	Year tuition fees eliminated	Expected age at enrollment	Earliest birth cohort exposed
*Treated countries*			
Cameroon	2000	6	1994
Ethiopia	1995	7	1988
Ghana	1996	6	1990
Kenya	2003	6	1997
Malawi	1994	6	1988
Rwanda	2003	7	1996
Uganda	1997	6	1991
Zambia	2002	7	1995
*Control countries*			
Benin	2006	6	2000 ^a^
Burkina Faso	2007	6	2001 ^a^
Burundi	2005	7	1998 ^a^
Lesotho ^b^	2006	6	2000 ^a^
Mozambique	2005	6	1999 ^a^
Namibia	2013	6	2007 ^a^
Tanzania	2002	7	1995 ^a^
Zimbabwe	-	7	none

^a^ Women born in this year or later are not included in currently available DHS data.

^b^ Lesotho’s Free Primary Education program began in 2000 and was phased in until 2006. We considered Lesotho tuition-free from 2006 onward.

Data on educational attainment and age at first marriage were obtained from Demographic and Health Surveys (DHS) conducted in each country between 1987 and 2014, the most recent survey year for which data were available at the time of this study. The DHS are repeated cross-sectional surveys that collect information on a wide range of sociodemographic and health variables. In most countries, DHS sample selection is based on a pre-existing sampling frame that lists all households in the country and is often the same list used to conduct recent censuses. Samples are usually stratified by geographic region and by urban and rural locations and are representative of the national population. The surveys use standardized questionnaires that make data comparable across countries and over time. Many countries have conducted multiple DHS, often at approximately five-year intervals. Further details on sampling methodology is available from the DHS Program [22]

All female respondents were asked to report their marital status and their age at the time they were first married or began living with a partner as if married. Using this information, we created two binary variables indicating whether a woman was married before 15 or before 18 years of age. Women who had never been married were considered not married as children and included in the denominator of our prevalence estimates. A total of six women who reported that they had been married were missing information on their age at the time of their first marriage and these women were dropped from our analyses.

All respondents were also asked to report the highest level of schooling they attended (i.e. primary, secondary) and the highest standard within that level that they completed. Women were considered to have completed primary school if they reported completing the highest standard of primary school in their country of residence or if they reported attending secondary school or a higher level. Fewer than 1% of respondents were missing data on their educational attainment (n = 3,935) and over a third of those missing values for this variable (n = 1,225) were included in the 1988 Kenya DHS. These observations were not included in our analyses of the effects of policy change on primary school completion.

Although the DHS are cross-sectional, they include female respondents from a wide age range, usually 15–49 years, which permits measurement of trends over birth cohorts. We pooled data from all standard DHS waves conducted between 1987 and 2014 in each country to create a dataset that included women born between 1970 and 2000. We then defined exposure to tuition-elimination policies as a function of birth cohort and country of residence. Women who reached the expected age of entry to primary school in the same year the policy was implemented or later were considered exposed to tuition-free schooling, as detailed in Table 1. For example, Uganda eliminated primary school tuition fees in 1997. Children in Uganda are expected to begin primary school at 6 years of age and therefore, respondents born in 1991 or later were considered exposed to the policy because they were expected to enroll in primary school in the same year the policy was implemented (or later) and to receive the entire duration of their primary schooling tuition-free. Eight of the 16 countries in our analysis had conducted a DHS recently enough for exposed women to be included in survey samples. We refer to these countries as “treated” throughout the manuscript. However, with the exception of Zimbabwe, all of the control countries eventually eliminated tuition fees as well, but did so too recently for any exposed women to have reached the age of 15 and be picked up in currently available DHS data.

All women interviewed between 15–49 years of age were used to estimate the effect of policy change on marriage before the age of 15. In order to avoid problems with censoring, women interviewed before the age of 18 were excluded from the sample used to estimate the effects of policy change on the probability of completing primary school and of marriage before the age of 18. Although children are expected to complete primary school between the ages of 13 and 15 in each of the countries included in our analysis, late entry and grade repetition are common in many countries, meaning that many older adolescents are still enrolled in primary school. All of the exposed women in Cameroon and Kenya were interviewed between the ages of 15 and 17 and so these two countries are considered treated only when examining the effect of the policy on marriage before the age of 15. DHS waves used and sample sizes in each country are listed in Table 2.

**Table 2:** Sample size and DHS waves used in each country included in the analysis. Exposed women are those born in cohorts affected by legislative changes. Unexposed women were born in cohorts unaffected by tuition-elimination policies.

		Interviewed between 15–49 years		Interviewed between 18–49 years	
Country	DHS waves used	Exposed	Unexposed	Exposed	Unexposed
*Treated countries*					
Cameroon^1^	1991, 1998, 2004, 2011	1,719	23,953	-	20,597
Ethiopia	2000, 2005, 2010	7,618	27,079	3,634	24,586
Ghana	1988, 1993, 1998, 2003, 2008, 2014	4,119	16,592	2,408	14,403
Kenya^1^	1988, 1993, 1998, 2003, 2008, 2014	3,371	46,178	-	40,681
Malawi	1992, 2000, 2004, 2010	8,341	30,837	4,060	28,089
Rwanda	1992, 2000, 2005, 2010, 2014	2,440	38,951	624	32,959
Uganda	1988, 1995, 2000, 2006, 2011	2,620	20,853	1,072	17,289
Zambia	1992, 1996, 2001, 2007, 2013	2,901	30,227	733	25,826
*Control countries*					
Benin	1996, 2001, 2006, 2011	-	35,145	-	30,195
Burkina Faso	1992, 1998, 2003, 2010	-	28,719	-	23,029
Burundi	1987, 2010	-	8,554	-	6,746
Lesotho	2004, 2009, 2014	-	17,463	-	14,342
Mozambique	1997, 2003, 2011	-	25,136	-	20,552
Namibia	1992, 2000, 2005, 2013	-	21,703	-	17,608
Tanzania	1991, 1996, 1999, 2004, 2009	-	25,407	-	19,755
Zimbabwe	1988, 1994, 1999, 2005, 2010	-	22,088	-	17,188
**TOTAL**		**33,129**	**418,885**	**12,531**	**353,845**

^1^ Cameroon and Kenya are considered treated countries only when using all women interviewed between 15–49 years of age.

### Statistical analyses

We used a difference-in-differences approach that exploits variation in the timing of policy implementation between countries to estimate the effect of eliminating tuition fees on the probability of completing primary school and of being married before 15 or 18 years of age [23]. This method allowed us to identify the effect of the policy while controlling for trends in each of the outcomes that were present before the policies were implemented. Specifically, we used logistic regression models of the following form:

$$logit\;Y_{i,c,t}=\beta_{0}+\beta_{1}({policy}_{c,t})+\gamma_{c}+\delta_{t}$$

In this model *logit Y_i,c,t_* represents the log odds of primary school completion or marriage before the specified age for woman *i* living in country *c* and born in year *t*. The variable *policy_c,t_* is an indicator equal to one if women living in country *c* and born in year *t* were exposed to tuition-elimination policies and the coefficient *β*_1_ identifies the treatment effect. The variables *γ_c_* and *δ_t_* represent fixed effects for country and birth cohort that control for time-invariant differences between countries and underlying trends in each of the outcomes shared across countries, respectively. We used the coefficients obtained from the logistic regression to estimate the marginal probability of each outcome among exposed and unexposed women in the post-policy period and subtracted these estimates to calculate risk differences. Standard errors were adjusted to account for clustering by country [24].

The validity of our estimates rests upon the assumption that trends in each of the outcomes observed among control countries are a reasonable representation of the trends that would have been observed in the treated countries had they not eliminated tuition fees or had they done so at a later point in time. We evaluated the tenability of this assumption in three ways. First, we graphed trends in the prevalence of primary school completion and child marriage among women born between 1970 and 1987, the “pre-policy” period, and looked for visual evidence of differences in trends between treated and control countries. We also estimated the average annual change in each outcome over this time period in each country and looked for outliers. Finally, we regressed country-level treatment status on each of the outcomes and included a product term between country-level treatment status and birth cohort to look for statistically significant differences in trends between treated and control countries over the pre-policy period.

After estimating the effect of policy change among the pooled sample, we also estimated the effect in each treated country. To do this, we compared each treated country individually to pooled data from all of the control countries using the same difference-in-differences methodology described above. In these estimates, data from all other treated countries was dropped.

All of our estimates are weighted using de-normalized sampling weights, following guidelines for the use of pooled data provided by the DHS Program [22]. Analyses were conducted using Stata 14 statistical software [25].

## Results

### Examination of pre-policy trends

**Fig 1:**
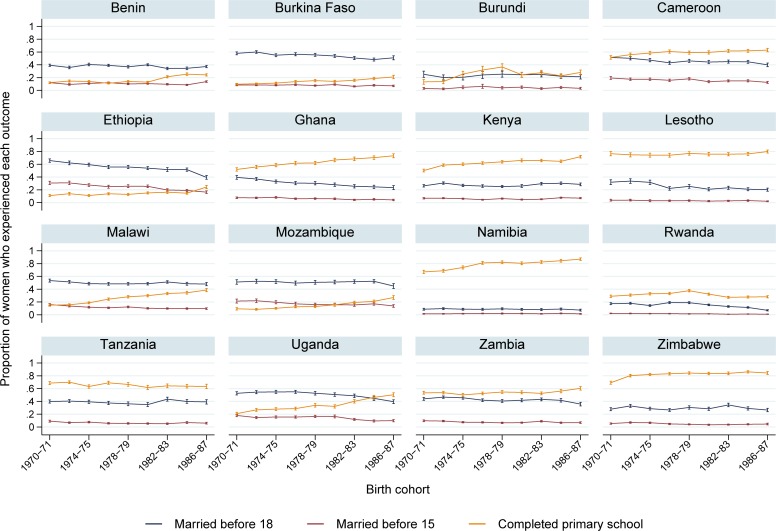
Pre-treatment trends in primary school completion and the prevalence of marriage before 15 and 18 years of age in each country.

Trends in each of the outcomes among women born during the pre-policy period from 1970–1987 are shown in Fig 1. There is little visual evidence of change in the prevalence of marriage before the age of 15 over this period in most of the countries, a result consistent with previous work that found slow progress toward the reduction of marriage among the youngest girls in the region over an even longer period [10]. Marriage before the age of 18 has fallen slowly but consistently in many countries. Primary school completion increased steadily throughout this period though levels vary widely between countries.

Estimates of the average annual change in each of the outcomes in each country are shown in the supporting information (S1 Table). As expected based on the results presented in Fig 1, annual changes in the percentage of women married before 15 and 18 years of age were small across the sample. The prevalence of marriage before either age changed by less than half a percentage point per year in most countries. The results of chi-squared tests further support our assertion that there are no substantial differences in pre-policy trends between treated and control countries.

### Effects on child marriage and primary school completion

The prevalence of marriage before the age of 15 declined by an average of 2 percentage points across all treated countries following the elimination of tuition fees (Table 3). Declines were larger in Kenya, Rwanda, Uganda and Zambia, where the prevalence of marriage among the youngest girls fell by 4–5 percentage points. Marriage before the age of 18 also declined by an average of 3 percentage points in our pooled analysis, though this estimate is less precise than that for marriage before the age of 15 and obscures notable variation between countries. The prevalence of marriage before the age of 18 declined by 10 percentage points in Ethiopia and by 15 percentage points in Rwanda following policy implementation. We found no evidence that the policies had any effect on child marriage in Cameroon or Malawi.

**Table 3:** Estimates of the effect of eliminating primary school tuition fees on the probability of completing primary school and of marriage before 15 or 18 years of age among the pooled sample and in individual countries.

Country	Marriage before 15 RD (95% CI)	Marriage before 18 RD (95% CI)	Primary school completion RD (95% CI)
**All countries**	-0.02 (-0.03, -0.01)	-0.03 (-0.09, 0.03)	0.01 (-0.03, 0.06)
Cameroon	-0.01 (-0.03, 0.01)	**-**	**-**
Ethiopia	-0.03 (-0.04, -0.02)	-0.10 (-0.13, -0.08)	0.03 (-0.01, 0.08)
Ghana	-0.00 (-0.02, 0.02)	-0.06 (-0.08, -0.04)	0.04 (-0.00, 0.08)
Kenya	-0.05 (-0.05, -0.04)	**-**	**-**
Malawi	-0.01 (-0.02, -0.00)	0.02 (-0.00, 0.05)	0.00 (-0.05, 0.05)
Rwanda	-0.05 (-0.07, -0.04)	-0.15 (-0.17, -0.13)	0.19 (0.17, 0.22)
Uganda	-0.04 (-0.05, -0.03)	-0.07 (-0.11, -0.04)	-0.01 (-0.06, 0.05)
Zambia	-0.04 (-0.05, -0.02)	-0.05 (-0.06, -0.03)	0.06 (0.04, 0.08)

RD: Risk Difference, CI: Confidence Interval

We hypothesized that some of the observed variation between countries in the effect of these policy changes on child marriage may be explained by differences in the effectiveness of the policies at improving educational attainment. The probability of completing primary school was increasing in most countries prior to the removal of tuition fees (Fig 1). Our results indicate that the implementation of these policies did not lead to additional gains in primary school completion in Malawi or Uganda and had modest effects on completion rates in Ethiopia and Ghana. We estimate that the probability of completing primary school rose by 19 percentage points in Rwanda and by 6 percentage points in Zambia following tuition fee removal.

## Discussion

Our pooled estimates indicate that the average effect of eliminating primary school tuition fees was modest across all treated countries. However, country-specific estimates indicate that the effect of these policy changes differed substantially across countries. Six of the eight treated countries experienced declines in the prevalence of child marriage following the policy change. These declines were concentrated among older adolescents in Ethiopia, Ghana, and Rwanda, were reductions in marriage before the age of 18 were markedly greater than those in marriage before the age of 15.

Differences in the effectiveness of tuition-fee removal policies at improving access to primary schooling across countries could plausibly account for some of the heterogeneity we observe in the magnitude of effects on child marriage. However, with the exception of Rwanda and Zambia, declines in child marriage were not accompanied by large increases in the probability of primary school completion. It is important to note that these are national-level estimates and that the removal of tuition fees likely had differential effects within countries. Tuition fees are more likely to be a barrier to enrolment for students from poor families. Previous work has shown that the greatest increases in enrollment were found among students from poor and rural backgrounds in Uganda, though results from Kenya are mixed [17,26,27]. Girls from poor families are also more likely to be married as children and it is possible that the elimination of tuition fees had a positive effect on the attainment of poorer girls but no effect on the attainment of girls from relatively wealthier families, leading to null net effects on schooling but declines in child marriage on a national scale. Moreover, the policies may have led to increases in the number of years that students attended primary school even if they did not have a measurable effect on primary school completion. In a pre-post evaluation of Uganda’s fee removal policy, Nishimura et al. (2008) found that students were more likely to complete primary standards 4 and 5 after the policy was implemented but that the difference diminished at higher standards [28]. Such increases in the duration of schooling could plausibly lead to delays in marriage without corresponding primary school completion rates.

Our analysis relies on a number of assumptions that warrant careful consideration. First, our definition of exposure assumes that girls enrolled in primary school at the expected age of entry. Some children enroll late, though little data is available to quantify the extent of delayed enrollment in most low-income countries. We were unable to determine the age at which respondents began primary school using DHS data. Older girls who enrolled in primary school following the removal of tuition fees would have been considered unexposed in our analysis, potentially leading to an underestimate of the effect of removing tuition fees.

The inclusion of fixed effects for country and birth cohort help control for unmeasured baseline differences between countries that could affect the outcomes, for changes in variables relevant to the outcomes shared across countries over time, such as trends in economic development, and for pre-existing trends in each of the outcomes. However, our estimates could still be confounded if other policies that influence child marriage were implemented at exactly the same point in time in any given country. Many of the countries included in our analysis adopted or modified minimum-age-at-marriage laws between the 1990s and mid 2000s and these policy changes could have affected child marriage rates over the time period examined in this study. We obtained information on the timing of these legislative changes from the WORLD Policy Analysis Center at the University of California, Los Angeles and determined that in every country, changes to minimum-age-at-marriage laws affected women born in earlier cohorts than those affected by tuition-free schooling policies. Therefore, depending on the precise timing of changes to minimum-age-at-marriage laws, any resulting change in the prevalence of child marriage was either included in the pre-policy trends shown in Fig 1 or would have been visible as a discontinuity in those trends. We see no visual evidence of shocks in the prevalence of child marriage during the pre-policy period from 1970–1987, and additional research has shown that these laws have not led to substantial changes in child marriage rates, largely due to lack of enforcement [29]. Moreover, the fact that our analysis includes multiple treated countries that eliminated tuition fees at different points in time limits the extent to which an unknown policy change in any single country could affect our pooled estimates.

In contrast to earlier randomized interventions, we studied the rollout of large scale policy initiatives under real world conditions. Challenges associated with the implementation of these policies are well documented. In many countries, tuition fees were eliminated without corresponding investment in infrastructure or increases in the number of qualified teachers, leading to severe classroom overcrowding, large student-to-teacher ratios, and concerns regarding the quality of education available following massive increases in enrollment [16,17,27,30,31]. We find that the policies led to reductions in child marriage despite these difficulties but gains may be offset over time if the perceived returns to schooling decline. Our work provides further support for improving educational opportunities as a mechanism for preventing child marriage and evaluates a specific national-level policy intervention that can be leveraged to this effect. We may come closer to eliminating child marriage and to attaining a host of other public health and development goals as the quality of the education available is strengthened.

## Supporting information

S1 Table The percentage of women born during the pre-policy period between 1970 and 1987 who experienced each outcome and estimates of the average annual percentage-point change in each outcome over the same time period. (DOCX)
