# Supplementary material for: The impact of eliminating primary school tuition fees on child marriage in sub-Saharan Africa: A quasi-experimental evaluation of policy changes in 8 countries
Source: PLoS One. 2018 May 24;13(5):e0197928. doi: 10.1371/journal.pone.0197928 (PMC5967724; doi:10.1371/journal.pone.0197928)
Supplement: S1 Table — (DOCX) [file pone.0197928.s001.docx]

**Supplementary Table 1. The percentage of women born during the pre-policy period between 1970 and 1987 who experienced each outcome and estimates of the average annual percentage-point change in each outcome over the same time period.**

| **Country** | **Marriage before 15 years of age** | | **Marriage before 18 years of age** | | **Primary school completion** | |
| --- | --- | --- | --- | --- | --- | --- |
|  | % married | Avg. annual change | % married | Avg. annual change | % completed | Avg. annual change |
| *Treated countries* | | | | | | |
| Cameroon | 16 | -0.3 | 46 | -0.3 | 59 | 0.4 |
| Ethiopia | 24 | -0.9 | 55 | -1.1 | 14 | 0.6 |
| Ghana | 6 | -0.3 | 30 | -0.8 | 62 | 1.3 |
| Kenya | 6 | 0.1 | 28 | 0.3 | 63 | 0.6 |
| Malawi | 11 | -0.3 | 50 | 0.0 | 27 | 1.3 |
| Rwanda | 2 | -0.1 | 15 | -0.5 | 31 | -0.3 |
| Uganda | 14 | -0.4 | 51 | -0.7 | 33 | 1.5 |
| Zambia | 8 | -0.2 | 43 | -0.5 | 54 | 0.3 |
| *Control countries* | | | | | | |
| Benin | 11 | 0.0 | 38 | -0.2 | 16 | 0.8 |
| Burkina Faso | 8 | 0.1 | 55 | -0.6 | 14 | 0.5 |
| Burundi | 4 | 0.1 | 23 | 0.0 | 25 | 0.7 |
| Lesotho | 3 | -0.1 | 25 | -0.7 | 76 | 0.3 |
| Mozambique | 17 | -0.4 | 51 | 0.0 | 14 | 1.1 |
| Namibia | 2 | 0.0 | 9 | -0.1 | 78 | 1.3 |
| Tanzania | 7 | -0.2 | 39 | -0.4 | 66 | -0.2 |
| Zimbabwe | 5 | -0.2 | 29 | -0.1 | 82 | 0.7 |
| All treated countries | 12 | -0.3 | 43 | -0.6 | 35 | 0.7 |
| All control countries | 8 | -0.1 | 38 | -0.2 | 53 | 0.2 |
| $\chi^{2}$ test for differences in trend | p=0.39 | | p=0.11 | | p=1.00 | |
